# A Comprehensive Comparison of Clinical Presentation and Outcomes of Kidney Transplant Recipients with COVID-19 during Wave 1 versus Wave 2 at a Tertiary Care Center, India

**DOI:** 10.1155/2022/9088393

**Published:** 2022-06-02

**Authors:** Sanjiv Jasuja, Gaurav Sagar, Anupam Bahl, Neharita Jasuja, Rajesh Chawla, Avdhesh Bansal, Manjit. S. Kanwar, Sudha Kansal, Nikhil Modi, Athar P. Ansari, Viny Kantroo, Purnima Dhar, Chitra Chatterjee, Nitin Ghonge, Samir Tawakley, Shalini Verma

**Affiliations:** ^1^Indraprastha Apollo Hospital, Department of Nephrology, New Delhi, India; ^2^AVATAR Foundation, Department of Clinical Research, New Delhi, India; ^3^Indraprastha Apollo Hospital, Department of Respiratory and Critical Care Medicine, New Delhi, India; ^4^Indraprastha Apollo Hospital, Department of Anaesthesia, New Delhi, India; ^5^Indraprastha Apollo Hospital, Department of Radiology, New Delhi, India; ^6^Apollo Hospital, Department of Nephrology, Noida, Uttar Pradesh, India

## Abstract

Data comparing the clinical spectrum of COVID-19 in kidney transplant recipients (KTRs) during the first and second waves of the pandemic in India is limited. Our single-center retrospective study compared the clinical profile, mortality, and associated risk factors in KTRs with COVID-19 during the 1st wave (1^st^ February 2020 to 31^st^ January 2021) and the second wave (1^st^ March-31^st^ August 2021). 156 KTRs with PCR confirmed SARS-CoV-2 infection treated at a tertiary care hospital in New Delhi during the 1st and the second waves were analyzed. The demographics and baseline transplant characteristics of the patients diagnosed during both waves were comparable. Patients in the second wave reported less frequent hospitalization, though the intensive care unit (ICU) and ventilator requirements were similar. Strategies to modify immunosuppressants such as discontinuation of antinucleoside drugs with or without change in calcineurin inhibitors and the use of steroids were similar during both waves. Overall patient mortality was 27.5%. The demographics and baseline characteristics of survivors and nonsurvivors were comparable. A higher percentage of nonsurvivors presented with breathing difficulty, low SpO_2_, and altered sensorium. Both wave risk factors for mortality included older age, severe disease, ICU/ventilator requirements, acute kidney injury (AKI) needing dialysis, Chest Computerized Tomographic (CT) scan abnormalities, and higher levels of inflammatory markers particularly D-dimer and interleukin-6 levels. *Conclusions*. KTRs in both COVID-19 waves had similar demographics and baseline characteristics, while fewer patients during the second wave required hospitalization. The D-dimer and IL-6 levels are directly correlated with mortality.

## 1. Introduction

Coronavirus disease 2019 (COVID-19), an infectious disease first identified in 2019 in Wuhan, China, has since spread worldwide [1–5]. The World Health Organization (WHO) declared the coronavirus outbreak a pandemic on 11th March 2020 [6]. COVID-19 is caused by severe acute respiratory syndrome coronavirus 2 (SARS-CoV-2) and is mainly spread by infected persons during close contact and via respiratory droplets produced when people cough or sneeze [1, 2, 7]. Infected individuals develop flu-like symptoms that include, but are not limited to, sore throat, fever, cough, runny nose, sneezing, loss of smell, fatigue, and shortness of breath [2, 8–10]. Severe cases display symptoms such as difficulty in breathing, persistent chest pain or pressure, and confusion. They can progress to a more severe and systemic disease characterized by pneumonia, Acute Respiratory Distress Syndrome (ARDS), sepsis and septic shock, multiorgan failure, including acute kidney injury (AKI), and cardiac and cerebrovascular injury with fatal outcomes [2, 8–10]. Age more than 60 years and underlying comorbidities such as diabetes, hypertension, cerebrovascular disease, cardiac disease, chronic lung disease, chronic kidney disease, immune suppression, and cancer are major risk factors associated with the severe form of COVID-19 [11–14].

According to WHO, as of 5^th^ November 2021, 248,467,363 confirmed cases of COVID-19, including 5,027,183 deaths, have been reported globally [15]. In India, the first case was detected on 30^th^ January 2020, and since then, the numbers have steadily increased; on 5^th^ November 2021, a total of 34,366,987confirmed COVID-19 cases, including 1,42,826 active cases, 33,763,104 cured/discharged individuals, and 4,61,057 deaths, were reported [16]. The pandemic spread in different countries across the world at different timelines and with varied intensity. In India, the first wave commenced in March 2020 with daily cases peaking in mid-September 2020 and finally declining in January 2021, whereas the second wave was observed from March 2021, peaking in April 2021 and showing a steady remission by August 2021 [17].

The COVID-19 pandemic has dramatically impacted all aspects of medicine, including the care of patients with immune-mediated kidney diseases and KTRs [18–33]. The use of immunosuppressive medications and the presence of multiple comorbidities puts KTRs at high risk of COVID-19 [29]. Studies reporting on the outcomes of COVID-19 in KTRs have demonstrated increased morbidity and mortality in transplant patients [18–34]. Our recent publication also reported a 27% mortality rate in KTRs with COVID-19, which increases to 44% in hospitalized patients and 100% in patients requiring ventilation [34]. Following the resurgence of COVID-19 in various countries, investigators have compared the epidemiology and disease outcomes between the first, second, and in some cases, third COVID-19 waves [21, 25, 33, 35–54]. However, data on the effects of the second wave of COVID-19 on KTR patients and its comparison with the first wave scenario is limited and reveals diverging results [21, 25, 45, 47, 53]. Currently, only one single-center study has been reported from India that has retrospectively investigated the impact of the first and second waves of COVID-19 on KTR; however, the study duration of the second wave was limited to 31^st^ May 2021 [25]. Here, we present a recent comparison between KTRs with SARS-CoV2 infections during India's two COVID-19 pandemic waves after the decline in the trajectory of second wave cases across the country. We have documented the differences and similarities observed in clinical outcomes and hospital management of KTRs with SARS-CoV2 infections between the first wave (1^st^ February 2020 to 31^st^ January 2021) and the second wave (1^st^ March 2021 till 31^st^ August 2021), focussing primarily on mortality, associated risk factors, and the impact of treatment options on the outcome.

## 2. Materials and Methods

### 2.1. Study Design and Population

A retrospective study on the effect of COVID-19 on KTRs in India, between the study period 1^st^ February 2020 and 31^st^ August 2021, was conducted at a tertiary care hospital in New Delhi, India. 156 KTRs (154 living and 2 deceased donors) identified with SARS-CoV2 real-time reverse transcription-polymerase chain reaction (RT-PCR) confirmed infection and treated as either out-patient or hospitalized were included in the analysis. The two waves of COVID-19 in India, the first wave from 1^st^ February 2020 to 31^st^ January 2021 and the second wave from 1^st^ March 2021 till 31^st^ August 2021, were analyzed separately. The study evaluated the clinical symptoms, risk factors, laboratory profile, disease management, and mortality rate in KTRs.

The present study is a retrospective post-COVID-19 kidney transplant recipient pooled data analysis which excludes any compromise of personal or medical information of the subject. The study was approved by the designated institutional authority of the host institution, Indraprastha Apollo Hospital, New Delhi, to carry out data analysis and publication of manuscript/manuscripts.

### 2.2. Clinical Management of COVID-19 in KTRs

Treatment and follow-up of all patients were according to the hospital's clinical protocol. COVID-19 infection was diagnosed as per the guidelines of the WHO [55, 56]. Patients with positive SARS-CoV-2 RT-PCR from nasopharyngeal or oropharyngeal swabs were considered laboratory-confirmed cases. The disease severity and assessment parameters were as per the Chinese Centre for Disease Control (China CDC) criteria [57]. KTRs with positive SARS-CoV-2 RT-PCR were identified as mild or severe and managed accordingly by a designated COVID-19 treating team in consultation with the treating nephrologist, as described before [34].

**Figure 1:**
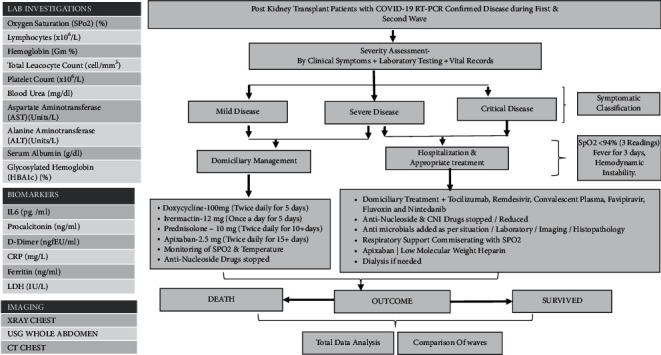
Schematic representation of clinical presentation, laboratory assessment, treatment options, and outcomes during the two waves of COVID-19 disease affecting kidney transplant recipients.

Patients were evaluated as per unit protocol (Figure 1) and were followed up for a minimum of 90 days (except in the case of a fatality).

### 2.3. Data Collection

Data were collected retrospectively from the medical records of the hospital or patients' follow-up submissions. Details of any asymptomatic home-isolated patients noncompliant with one or all prescribed drugs or investigation protocols were recorded and included in the study.

Collected data included demographics, transplantation history, comorbidities, concomitant medications, COVID-19-related symptoms, therapy during hospitalization, supportive measures needed during hospitalization, laboratory investigations (other than SARS-CoV-2 RT-PCR), and therapeutic outcomes (mortality and recovery). The onset symptom data were collected on first clinical reporting either by telephone for domiciliary patients or from triage notes for hospitalized patients. Based on the Body Mass Index criteria for the Asian population [58], the mean BMI was calculated.

The PCR test was repeated every 15 days until negative on two consecutive days.

### 2.4. Outcomes

The primary outcome of the study was to assess the mortality rate associated with COVID-19 in KTRs. The secondary outcomes included the spectrum of clinical presentation, immunosuppressive regimen, laboratory investigations, and pharmacological management of COVID-19 disease in the KTRs and their correlation with ICU admission, AKI, and acquired comorbidities (bacterial, fungal, or viral infections). AKI was defined using the Kidney Disease Improving Global Outcomes (KDIGO)-2012 [59] criteria with baseline serum creatinine. Chest CT scan done in patients with poor oxygen saturation levels regardless of the ongoing treatment was quantified based on the CT severity score index [60].

### 2.5. Statistical Analysis

The data was analyzed as described before [34]. Briefly, statistical analysis was performed on pooled data tabulated using Microsoft Excel, using the Statistical Package for Social Science (SPSS) version 16.0 (SPSS Inc., Chicago, IL). Continuous variables are represented as mean ± standard deviation (SD), and median and interquartile range (IQR) and qualitative variables are reported as numbers and percentages. For normally distributed variables, mean difference and 95% confidence intervals (CIs) were reported. For skewed variables, the median difference and its 95% CI were calculated using the Hodges Lehmann method; R-software version 3.6.1 was applied for determining the same.

Unpaired Student's *t*-test was performed to compare the mean between survivor and nonsurvivor groups for normally distributed variables having homogeneity of variance. The Welch test was applied when the homogeneity of variance between the groups was violated. For inflammatory markers and some biomarkers, the nonparametric Mann–Whitney *U* test was applied due to skewed distribution. The Chi-square and Fisher's exact test were applied to find the association between mortality and qualitative variables; the odds ratio and its 95% CI were reported.

To compare the discriminate power of biomarkers, Receiver Operating Characteristic (ROC) curve was applied. Multivariable logistic regression (MLR) to find the independent risk factors for nonsurvivors could not be performed due to the small number of cases, and some of the variables had zero count. MLR was performed to evaluate the independent effect of each biomarker on survivor status, adjusting age, hemoglobin (Hb), total leucocyte count (TLC), platelet count, blood urea, albumin level, fungal infection, chronic allograft dysfunction, and CAD/PVD. Bonferroni correction was applied, keeping into consideration the small sample size and multiple variable testing. The *p* value of less than 0.001 was considered statistically significant.

## 3. Results

### 3.1. Demographics, Comorbidities, and Baseline Transplant Characteristics of KTRs

156 KTRs with positive SARS-CoV-2 RT-PCR were included in the study, out of which 72 KTRs were from the 1^st^ wave of COVID-19 and 84 KTRs from the 2^nd^ wave.  Table 1 shows the demographics and comorbidities of the KTRs recorded at the time of presentation. The average age, weight, and height of the KTRs were 49.47 ± 13.1 years, 68.9 ± 14.99 kg, and 1.67 ± 0.09 meters, respectively. No significant difference was observed between the mean age, weight, height, median time interval from transplant to COVID-19, comorbidities, and baseline immunosuppressive regimens of the KTRs diagnosed during the 1^st^ wave or the 2^nd^ wave of the COVID-19 pandemic. Similarly, the mean BMI value was also comparable between the two waves. Notably, during both periods, the male to female ratio was skewed toward the male population; however, the difference between the gender distributions was more pronounced in the 2nd wave with male patients (Table 1).

### 3.2. Clinical Presentation

COVID-19 symptoms presented at the time of diagnosis are listed in Table 2. The major symptoms reported were fever (*n* = 140, 89.7%) and cough (*n* = 117, 75.0%); body ache (*n* = 77, 49.4%), sore throat (*n* = 53, 34.0%), and breathing difficulty (*n* = 48, 30.8%) were the other prominent complaints followed by distaste (*n* = 36, 23.1%), loose motion (*n* = 32, 20.5%), loss of smell (*n* = 22, 14.1%), running nose (*n* = 16, 10.3%), altered sensorium (*n* = 13, 8.3%), extreme weakness (*n* = 9, 5.8%), and incidental detection (*n* = 4, 5.6) (Table 2). Symptoms including sore throat (*n* = 35/84, 41.7% vs 18/72, 25%; *p* value .028), body aches (*n* = 50/84, 59.5% vs 27/72, 37.5%; *p* value .006), loss of smell (*n* = 18/84, 21.4% vs 4/72, 5.6%; *p* value .005), distaste (*n* = 26/84, 31% vs 8/72, 11.1%; *p* value .003), loose motions (*n* = 25/84, 29.8% vs 7/72, 9.7%; *p* value .002), and running nose (*n* = 15/84, 17.9% vs 1/72, 1.4%; *p* value .001) were reported more frequently during the second wave.

### 3.3. Clinical Outcome and Hospital Management

Details of clinical outcomes and treatment modalities of KTRs with COVID-19 are summarized in Table 3. Out of 156 KTRs included in the study, 78 (50%) were hospitalized and 78/156 patients with mild COVID-19 symptoms (50%) remained domiciliary. Less frequent hospitalization was observed during the second wave than during the first wave (*n* = 34/84, 40.5% vs *n* = 44/72, 61.1%, *p* value 0.01). However, patients requiring room air management, oxygen, ventilators, and ICU stay were comparable between the first and the second wave cohorts.

Immunosuppressive treatment regimens were modified in the majority of patients during both waves. In 128 (82.0%) patients, antinucleoside drugs were stopped, whereas in 5 (3.2%) patients, the dose was reduced, and in 19 (12.1%) patients, the treatment was continued as before; four patients (2.6%) were not taking antinucleoside drugs, to begin with. The antinucleoside drugs were stopped in more patients during the second wave than during the first wave (*n* = 75/84, 89.3% vs *n* = 53/72, 73.6%, *p* value 0.011).

CNIs remained unchanged in most patients (*n* = 116, 74.4%), and the administration was stopped in 22.5% (*n* = 36) patients; only 2 (1.3%) patients underwent a dose reduction of CNIs. The CNI drug treatment was altered in more patients during the first wave; however, the difference was not statistically significant.

Other specific treatments included the use of steroids (*n* = 156, 100%), ivermectin (*n* = 105, 67.3%), doxycycline (*n* = 102, 65.8%), remdesivir (*n* = 45, 38.8%), azithromycin, (*n* = 67, 42.9%), favipiravir (*n* = 53, 34%), fluvoxin (*n* = 44, 28.2%), convalescent plasma (*n* = 32, 20.5%), nintedanib (*n* = 8, 15.3%), tocilizumab (*n* = 10, 6.4%), HCQS (*n* = 9, 5.8%), antibiotics (*n* = 77, 49.4%), and antifungals (*n* = 33, 21.2%). Frequency of patients treated with steroids (100%), Azithromycin (*n* = 30/72, 41.7% vs 37/84, 44%, *p* value 0.765), remdesivir (*n* = 24/72, 33.3% vs 21/84, 25%, *p* value 0.252), HCQS (*n* = 7/72, 9.7% vs 2/84, 2.4%, *p* value 0.082), nintedanib (*n* = 1/72, 1.5% vs 7/84, 8.4%, *p* value 0.075), and antifungals (*n* = 18/72, 25% vs 15/84, 17.9%, *p* value 0.276) during both the waves were statistically comparable. During the second wave, fewer patients were treated with tocilizumab (*n* = 9/72, 12.5% vs 1/84, 1.2%, *p* value 0.004), convalescent plasma (*n* = 22/72, 30.6% vs 10/84, 11.9%, *p* value 0.004), and antibiotics (*n* = 42/72, 58.3% vs 35/84, 41.7%, *p* value 0.038) compared to administration of ivermectin (*n* = 40/72, 55.6% vs 65/84, 77.4%, *p* value 0.004) and doxycycline (*n* = 38/72, 53.5% vs 64/84, 76.2%, *p* value 0.003) although the observed differences were not found statistically significant. Notably, only patients from second wave were treated with antivirals favipiravir (*n* = 0/72, vs 53/84, 63.1%, *p* value <0.001) and fluvoxin (*n* = 0/72, vs 37/84, 52.4%, *p* value <0.001).

128/156 patients were also treated for Thromboprophylaxis by means of either antiplatelet treatment (*n* = 3, 1.9%), or low molecular weight heparin (LMWH) (*n* = 52, 33.3%), or oral anticoagulants (OAC) (*n* = 74, 47.4%). Significant differences were observed in Thromboprophylaxis treatment between both waves; LMWH treatment was preferred during the first wave (*n* = 31/72, 43% vs 21/84, 25%) compared to OAC (*n* = 20/72, 27.8% vs 54/84, 64.4%), which was used more during the second wave. Out of the 27 (17.3%) patients not treated for Thromboprophylaxis, the majority were in the first wave (*n* = 19/72, 26.4% vs *n* = 8/84, 9.5%).

AKI was observed in 65 (41.7%) patients, out of which 25 (16%) patients needed dialysis support. The frequency of patients with AKI and that of patients with AKI that needed dialysis were comparable between the two waves.

CT scan of the chest was performed on 67 patients that showed poor oxygen saturation levels despite ongoing treatment. CT findings were quantified based on the CT severity score index. Out of 67 patients, 23 (34.3%) had a CT score <10, 10 (14.9%) had a CT score 11–14, and 34 (50.7%) had a CT score ≥15. Patients that underwent a CT scan were higher during the second wave (*n* = 36 vs *n* = 31). However, the distribution of patients across the CT severity score index was comparable between the two waves.

### 3.4. Mortality in COVID-19-Infected KTRs and Comparison of Risk Factors for Mortality in the Two Waves

The overall patient mortality rate observed was 27.5% [95% CI: 20.7–35.2] (43/156). A detailed comparison of the demographics, immunosuppression regimen, clinical profile, treatment, clinical outcomes, and possible risk factors for mortality between survivors and nonsurvivors is summarized in Tables 4 and 5.

No significant difference was observed between survivors and nonsurvivors with regard to gender, blood group, BMI, and comorbidities (Table 4).

At the time of diagnosis, the frequencies of surviving and nonsurviving patients presenting COVID-19-related symptoms such as fever, cough, sore throat, body aches, loss of smell, distaste, loose motion, and extreme weakness were comparable. However, significantly higher percentage of nonsurvivors, compared to surviving patients, presented with symptoms of breathing difficulty (*n* = 24/43, 55.8% vs *n* = 24/113, 21.2%, *p* = 0.001) with low SpO_2_ (87.74 ± 7.82 vs 95.47 ± 3.36, *p* < 0.001) and altered sensorium (*n* = 13/43, 30.8% vs *n* = 0/113, 0%, *p* < 0.001) (Tables 4 and 5).

Significantly higher percentage of nonsurviving patients required a ventilator (*n* = 26/43, 60.5% vs *n* = 0/113, *p* < 0.001) and an ICU stay (*n* = 37/43, 86% vs *n* = 12/113, 10.6%, *p* < 0.001). Incidence of AKI (*n* = 36/43, 83.7% vs *n* = 29/113, 25.7%, *p* < 0.001) and requirement of dialysis support (*n* = 21/43, 48.8% vs *n* = 4/113, 3.5%, *p* < 0.001) were also significantly higher in nonsurvivors. Statistically significant risk factors that were observed in nonsurvivors included older age (*p* = 0.001), anemia (*p* < 0.001), low platelet count (*p* < 0.001), higher total leucocyte count (*p* < 0.001), kidney dysfunction as diagnosed by elevated serum creatinine (*p* < 0.001) and blood urea (*p* < 0.001), and higher levels of inflammatory markers, such as IL-6 level (*p* < 0.001), procalcitonin (*p* < 0.001), D-dimer (*p* < 0.001), CRP (*p* < 0.001), Ferritin (*p* < 0.001), LDH (*p* < 0.001), and CT score >15 (*p* < 0.001).

The impact of each biomarker on the survival status of KTRs as evaluated by multivariate logistic regression (MLR) analysis is summarized in Table 6. Only D-dimer and IL6 rise correlated with an increase in mortality; interestingly, every 5-unit increase in IL6 level increased the odds of mortality risk by 2.4% (Table 6).

**Figure 2:**
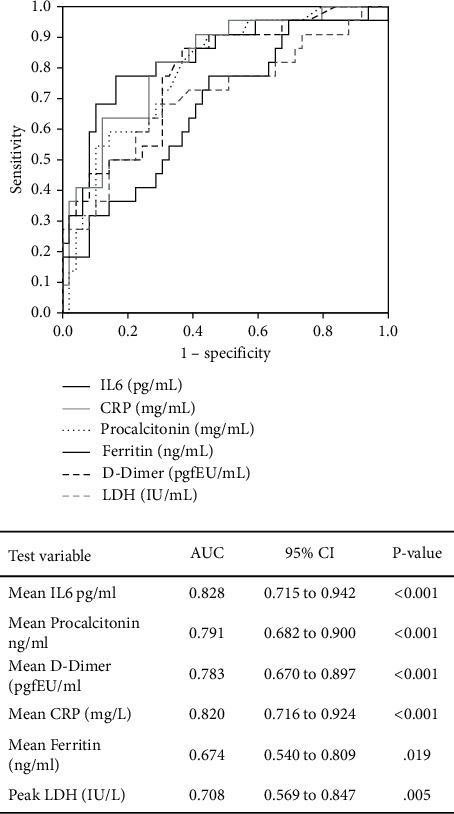
ROC curve and area under the curve (AUC) for biomarkers IL6, Procalcitonin, D-dimer, CRP, Ferritin, and LDH in KTRs with COVID-19.

Additional Receiver Operating Characteristic (ROC) curves were performed to determine the diagnostic values of inflammatory markers; all inflammatory biomarkers were found to be significant for diagnostic purposes (Figure 2). Furthermore, the area under the curve (AUC) was calculated to compare the different classifiers. For purposes of medical diagnosis, AUC values between 0.9 and 1, 0.8–0.9, 0.7–0.8, 0.6–0.7, and 0.5–0.6 were considered excellent, good, fair, poor, and failed, respectively [61]. Based on this classification, IL-6 and CRP were the most acceptable diagnostic markers, followed by procalcitonin and D-dimer (Figure 2).

## 4. Discussion

We have detailed a retrospective analysis comparing clinical outcomes and hospital management of 156 KTRs with confirmed COVID-19 between the first wave (1^st^ February 2020 to 31^st^ January 2021) and the second wave (1^st^ March 2021 till 31^st^ August 2021) of the pandemic. We identified 72 KTRs during the 1^st^ wave and 84 KTRs during the 2^nd^ wave. In contrast to a similar study by Kute et al. [25], our patient cohort included both domiciliary patients exhibiting milder symptoms of COVID-19 and hospitalized patients with moderate to severe COVID-19 symptoms. The demographics (age, weight, height, BMI, and blood group distribution) of the patients were comparable between the two waves. Interestingly, as reported previously [34], we did observe a male predominance in the patient cohort, the difference being more pronounced in the 2^nd^ wave.

No significant differences in terms of comorbidities, oxygen/ventilator requirement, ICU stay, the incidence of AKI (with or without the need for dialysis), and chest CT severity score index were observed between the first and the second wave cohorts. Similar to a previous report [25], we observed more mild COVID-19 cases that did not require hospitalization during the second wave.

The baseline immunosuppressive regimens comprising steroids and CNI were comparable between the patients from both waves. The consensus regarding the susceptibility of KTRs in developing severe COVID-19 is that immunosuppressive treatment impairs the immune response [19, 24, 28]. Treatment guidelines documented in the literature suggest modification of immunosuppression for better COVID-19 management [62]. In our study, immunosuppressive treatments were modified in the majority of patients during both waves in conjunction with other treatment options [63]. The antinucleoside drugs were stopped in more patients during the second wave (89.3% vs 72%) whereas CNI drug treatment was altered for more patients during the first wave.

According to a recent study [25], the use of HCQS and tocilizumab decreased, and that of dexamethasone and remdesivir increased during the second wave. Although not statistically significant, we also observed a decrease in the use of tocilizumab, convalescent plasma, and antibiotics and an increase in treatment with ivermectin and doxycycline during the second wave. Prescriptions for antivirals favipiravir and fluvoxin were administered only during the second wave. The variation between the preferred treatment options in our study and the previous report [25] could be explained by the difference in the patient cohort; our study population included both domiciliary and hospitalized patients while the previous study [25] mainly focussed on hospitalized patients. The modification in treatment made during the second wave could be a result of recent studies demonstrating the efficacies of investigational treatments or drugs that were tried during the first wave, and the introduction of new therapies, thereby providing empirical data for deciding which treatment to follow [1].

During the second wave of COVID-19, India was challenged with the emergence of coronavirus disease-associated mucormycosis in both active and recovered patients, which contributed significantly to the increase in morbidity and mortality of COVID-19 [54, 64]. In the present study, during the 2^nd^ wave, 3 KTRs with COVID-19 developed mucormycosis, while during the 1^st^ wave, only one patient reported the same. Notably, even though the overall use of antifungal drugs was comparable in the two waves, documented culture positive fungal infections were higher among nonsurvivors (*p* = 0.015).

In our study, we observed an overall patient mortality rate of 27.5% (43/156), similar to results reported previously by us [34] as well as studies conducted across several countries [12, 19, 21–23, 28, 30, 45, 65–70]. Mortality was significantly associated with ventilator requirement, ICU admission, the incidence of AKI, and the requirement of dialysis support.

The distribution of survivors and nonsurvivors as to gender, BMI, comorbidities, and blood group was comparable; however, as reported previously [34], the frequency of nonsurvivors with blood group A was higher (14/66; 38.8%). Studies have shown that individuals with blood group A were susceptible to developing the disease with unfavorable outcomes [71–73], possibly due to a lack of anti-A antibodies that have been shown to provide protection against SARS-COV-2 viral infection [74]. Additionally, the blood group A is linked to higher susceptibility of comorbidities that contribute to high mortality of severe COVID-19 [75, 76].

Notably, the nonsurvivors significantly presented breathing difficulty with low SpO2 and altered sensorium, supporting that KTRs with severe COVID-19 presentation were more susceptible to mortality.

Previous studies [12, 19, 21–23, 28, 30, 45, 65–70] have reported older age, anemia, low platelet count, higher total leucocyte count, kidney dysfunction as diagnosed by elevated serum creatinine, and blood urea, and CT score >15 and increased inflammatory markers (IL-6, procalcitonin, D-dimer, CRP, Ferritin, and LDH) as risk factors for mortality, which are also present in our study. Our analysis further shows that the increase in D-dimer and IL6 levels correlate with an increase in mortality and every 5-unit increase in IL6 levels increases mortality risk by 2.4%.

A comparison between the present work and similar studies from India as well as other countries [21, 25, 45, 47], detailing the similarities and differences between the work, is presented in Table 7. Despite the differences in geographical location and timeline of the epidemic waves, our study presents multiple similarities with other studies, especially with regard to milder symptoms and less hospitalization and COVID-19 treatment strategies during the second wave.

In summary, our study here provides a comprehensive comparison of the effect of COVID-19 on KTRs during the first and second waves of the disease outbreak in India, with relevance to mortality and risk factors associated with it. The inclusion of both hospitalized and home-isolated patients with milder symptoms in the total patient cohort allows us to provide a broader implication. In addition, the extended study period for the 2^nd^ wave (until 31^st^ August 2021) permitted us to include patients during the peak and remission of the second wave of the COVID-19 pandemic, thereby providing an inclusive patient cohort for analysis.

Our main limitation is that the study is a single-center study with a limited number of participants from a specific geographical location and therefore may not be sufficient to correctly represent the profile of the entire nation. Recent studies have also evaluated the effect of COVID-19 complications such as mucormycosis [54, 64], which adds more complexity to the treatment of KTRs. A multicentre study with a larger patient cohort, including a follow-up to study post-COVID-19 consequences, may not only validate our findings for the entire country but also promote awareness for better diagnosis and early management of post-COVID-19 complications.

## 5. Conclusions

In our patient cohort, combining both domiciliary and hospitalized individuals, we observed that the demographics and baseline transplant characteristics including the immunosuppressant regimen, comorbidities, requirement of ICU or ventilator, and incidence of AKI and radiological assessment by chest CT scan were similar between both waves. Interestingly, patients in the second wave reported less frequent hospitalization. Immunosuppressant treatments were modified during both waves as a strategy to build an immune response against the SARS-CoV-2 virus and treatment with antivirals favipiravir and fluvoxin was introduced in the second wave. Clinical symptoms such as breathing difficulty, low SpO2, and altered sensorium were presented at a higher rate in nonsurvivors. Common risk factors associated with mortality included older age, severe disease, ICU/ventilator requirements, acute kidney injury (AKI) needing dialysis, CT scan abnormalities, and higher levels of inflammatory markers particularly D-dimer and IL6 levels that correlated directly with mortality. Larger studies are needed to properly assess the outcomes of the second wave among KTRs and to address the potential use of IL6 and D-dimer as diagnostic biomarkers in identifying KTRs with severe COVID-19 disease.

## Figures and Tables

**Table 1:** Demographics, comorbidities, and baseline kidney transplant recipients (KTRs) characteristics at the time of diagnosis of COVID-19 in two waves of disease.

Characteristic	Classification	Total (*n* = 156)	Wave 1 (*n* = 72)	Wave 2 (*n* = 84)	*p* value
*Demographics*					
Age (years) (Mean ± SD)		49.47 ± 13.06	51.15 ± 13.0	48.04 ± 13.01	0.138
Height (meter) (Mean ± SD)		1.67 ± 0.09	1.67 ± 0.08	1.67 ± 0.09	0.661
Weight (kg) (Mean ± SD)		68.9 ± 14.99	70.66 ± 15.08	67.37 ± 14.83	0.127
Body Mass Index (kg/m^2^) (Mean ± SD)		24.67 ± 5.00	25.34 ± 5.27	24.09 ± 4.70	0.121
Gender, *n* (%)	Male	120 (76.9)	55 (76.4)	65 (77.4)	0.883
	Female	36 (23.1)	17 (23.6)	19 (22.6)	
Blood group, *n* (%)	O	36 (23.1)	22 (30.1)	14 (16.7)	0.032
	A	36 (23.1)	20 (27.4)	16 (19.0)	
	B	66 (42.3)	25 (34.7)	41 (48.8)	
	AB	18 (11.5)	5 (6.8)	13 (15.7)	

*Comorbidities, n (%)*					
Preexisting comorbidities	Diabetes Mellitus (DM)	86 (55.1)	37 (51.4)	49 (58.3)	0.385
	Hypertension (HTN)	140 (89.7)	65 (90.3)	75 (89.3)	0.839
	Chronic liver disease (CLD)	10 (6.4)	4 (5.6)	6 (7.1)	0.687
	Chronic obstructive airways disease (COAD)	13 (8.3)	8 (11.1)	5 (6.0)	0.245
	Vascular disease (CAD/PVD)	37 (23.7)	19 (26.4)	18 (21.4)	0.468
	Chronic allograft dysfunction	41 (26.3)	21 (29.2)	20 (23.8)	0.449
	Obstructive sleep apnoea (OSA)	7 (4.5)	4 (5.6)	3 (3.6)	0.703^$^
Acquired comorbidities	Cytomegalovirus (CMV) Activation	5 (3.2)	3 (4.2)	2 (2.4)	0.663^$^
	Mucormycosis	4 (2.6)	1 (1.4)	3 (3.6)	0.625^$^
	Fungal Culture Positivity^#^	9 (5.8)	1 (1.4)	8 (9.5)	0.039^$^
	Bacterial Blood Culture Positive	10 (6.4)	5 (6.9)	5 (6.0)	1.00^$^
	Bacterial Urine Culture Positive	5 (3.2)	4 (5.6)	1 (1.2)	0.182^$^

*KTRs baseline clinical characteristics*					
Transplant duration (weeks) median [25^th^-75^th^ percentile]		282 [123.6–425.9]	275.3 [131.8–406.4]	297 [116.7–462.4]	0.710
Baseline immunosuppression *n* (%)	CNI (Tac/CyA)	154 (98.7)	71 (98.6)	83 (98.9)	1.000^$^
	MMF/MPA	153 (98.1)	70 (97.2)	83 (98.9)	1.000^$^
	Steroids	156 (100)	72 (100)	84 (100)	1.000^$^

^$^: Fisher's exact test. CAD/PVD: Coronary Artery Disease/Peripheral Vascular Disease; CNI: calcineurin inhibitors; MMF: Mycophenolate Mofetil; Tac: Tacrolimus; CyA: cyclosporine A. ^#^Fungal Culture Positivity-when fungal infection was documented by positive urine or blood or body fluid culture.

**Table 2:** COVID-19 related symptoms in KTRs in both waves.

Symptoms	Total (*n* = 156)	Wave 1 (*n* = 72)	Wave 2 (*n* = 84)	*p* value
	*n* (%)	*n* (%)	*n* (%)	
Fever	140 (89.7)	62 (86.1)	78 (92.9)	0.166
Cough	117 (75.0)	51 (70.8)	66 (78.6)	0.266
Sore throat	53 (34.0)	18 (25.0)	35 (41.7)	0.028
Body aches	77 (49.4)	27 (37.5)	50 (59.5)	0.006
Breathing difficulty	48 (30.8)	27 (37.5)	21 (25.0)	0.092
Loss of smell	22 (14.1)	4 (5.6)	18 (21.4)	0.005
Distaste	36 (23.1)	10 (13.9)	26 (31.0)	0.012
Loose motions	32 (20.5)	7 (9.7)	25 (29.8)	0.002
Extremes weakness	9 (5.8)	6 (8.2)	3 (3.6)	0.203
Altered sensorium	13 (8.3)	7 (9.7)	6 (7.1)	0.561
Running nose	16 (10.3)	1 (1.4)	15 (17.9)	0.001^$^
Incidental	4 (2.6)	3 (4.2)	1 (1.2)	0.336^$^

Data represents the frequency distribution of the study population as *n*(%). ^$^Fisher's exact test.

**Table 3:** Clinical outcome and management of KTRs with COVID-19 in both waves

Parameters	Number (*n* = 156)	Percentage	Wave 1 (*n* = 72) *n* (%)	Wave 2 (*n* = 84) *n* (%)	*p* value
*Treatment parameters*					
Hospitalization	78	50.0	44 (61.1)	34 (40.5)	0.010
Domiciliary	78	50.0	28 (38.8)	50 (59.5)	0.010
Room air management	83	53.2	36 (50.0)	47 (56.0)	0.458
Oxygen with mask	29	17.3	16 (18.6)	13 (15.5)	0.280
Noninvasive ventilator	13	8.3	6 (8.3)	7 (8.3)	1.00
Ventilator	27	17.3	14 (19.4)	13 (15.5)	0.514
Steroid	156	100	72 (100)	84 (100)	1.00
Azithromycin	67	42.9	30 (41.7)	37 (44.0)	0.765
HCQS	9	5.8	7 (9.7)	2 (2.4)	0.082
Ivermectin	105	67.3	40 (55.6)	65 (77.4)	0.004
Doxycycline	102	65.8	38 (53.5)	64 (76.2)	0.003
Tocilizumab	10	6.4	9 (12.5)	1 (1.2)	0.006^$^
Remdesivir	45	38.8	24 (33.3)	21 (25.0)	0.252
Convalescent plasma	32	20.5	22 (30.6)	10 (11.9)	0.004
Favipiravir	53	34.0	0	53 (63.1)	<0.001^$^
Fluvoxin	44	28.2	0	44 (52.4)	<0.001^$^
Nintedanib	8	15.3	1 (1.5)	7 (8.4)	0.070^$^

*Thromboprophylaxis*					
Antiplatelet	3	1.9	2 (2.8)	1 (1.2)	<0.001^$^
LMWH	52	33.3	31 (43)	21 (25.0)	
OAC	74	47.4	20 (27.8)	54 (64.4)	
Not taking	28	17.9	20 (27.8)	8 (9.5)	

*Antinucleoside drugs*					
Continued	19	12.1	12 (16.7)	7 (8.3)	0.062^$^
Dose reduced	5	3.2	4 (5.5)	1 (1.2)	
Drug stopped	128	82.0	53 (73.6)	75 (89.2)	
Not taking	4	2.6	3 (4.2)	1 (1.2)	

*CNI drugs (tacrolimus or cyclosporine)*					
CNI continued	116	74.4	50 (69.4)	66 (78.6)	0.813
CNI dose reduced	2	1.3	1 (1.4)	1 (1.2)	
CNI stopped	36	22.5	20 (27.8)	16 (19.0)	
Not taking	2	1.3	1 (1.4)	1 (1.2)	

*AKI and need for dialysis support (CRRT/SLEDD/Intermittent hemodialysis)*					
Total AKI patients	65	41.7	29 (40.3)	36 (42.9)	0.745
AKI patients needing dialysis	25	16.0	13 (18.1)	12 (14.3)	0.522

*Computerized tomographic scanning with CT score (N* *=* *67)*			*N* = 31	*N * ** **= 36	
CT score ≤10	23	34.3	9 (29.0)	14 (38.9)	0.538
CT score 11–14	10	14.9	6 (19.4)	4 (11.1)	
CT score ≥15)	34	50.7	16(51.6)	18 (50.0)	

*Other outcomes*					
ICU requirement	49	31.4	22 (30.6)	27 (32.1)	0.864
Antibiotics used	77	49.4	42 (58.3)	35 (41.7)	0.038
Antifungal used	33	21.2	18 (25.0)	15 (17.9)	0.276

^$^ Fisher's exact test. HCQS: Hydroxychloroquine Sulfate; LMWH: low molecular weight heparin; AKI: Acute Kidney Injury; OAC: oral anticoagulants; CNI: calcineurin inhibitors; CRRT: Continuous Renal Replacement Therapy; SLEDD: Slow Low-Efficiency Daily Dialysis.

**Table 4:** Comparison between survivors and nonsurvivors.

Variable	Total (*n* = 156) *n* (%)	Survivor (*n* = 113) *n* (%)	Nonsurvivors (*n* = 43) *n* (%)	Odds ratio (95% CI)^*∗*^	*p* value
*Gender*					
Male	120 (76.9)	86 (76.1)	34 (79.1)	1.19 [0.50 to 2.79]	0.695
Female	36 (23.1)	27 (23.9)	13 (30.2)	1.0	

*Blood group*					
O	36 (23.1)	27 (23.9)	9 (20.9)	1.0	
A	36 (23.1)	22 (19.5)	14 (32.6)	1.91 [0.70–5.24]	0.209
B	66 (42.3)	48 (42.5)	18 (41.9)	0.38 [0.07–1.96]	0.245
AB	18 (11.5)	16 (14.2)	2 (4.7)	1.13 [0.44–2.85]	0.804

*Preexisting comorbidities*					
Diabetes mellitus (DM)	86 (55.1)	58 (51.3)	28 (65.1)	1.77 [0.86–3.66]	0.124
Hypertension (HTN)	140 (89.7)	98 (86.7)	42 (97.7)	6.43 [0.82–50.25]	0.076
Chronic liver disease (CLD)	10 (6.4)	7 (6.2)	3 (7.0)	1.14 [0.28–4.61]	1.00
Chronic obstructive airways disease (COAD)	13 (8.3)	9 (8.0)	4 (9.3)	1.18 [0.35–4.07]	0.754
Vascular disease (CAD/PVD^*@*^)	37 (23.7)	20 (17.7)	17 (39.5)	3.04 [1.40–6.63]	0.004
Chronic allograft dysfunction	41 (26.3)	25 (22.1)	16 (37.2)	2.09 [0.97–4.47]	0.056
Obstructive sleep apnoea (OSA)	7 (4.5)	5 (4.4)	2 (4.8)	1.08 [0.20–5.79]	1.00

*Acquired comorbidities*					
Cytomegalovirus (CMV) Activation	4 (2.6)	0 (0.0)	4 (9.3)	—	0.005^$^
Fungal Culture Positivity #	9 (5.8)	3 (2.7)	6 (14.0)	5.95 [1.42–24.97]	0.015
Bacterial Blood Culture Positivity	10 (6.4)	1 (0.9)	9 (20.9)	29.65 [3.63–242.42]	<0.001
Bacterial Urine Culture Positivity	5 (3.2)	1 (0.9)	4 (9.3)	11.49 [1.25–105.9]	0.021

*Baseline immunosuppression*					
CNI (Tac/CyA)^@^	154 (98.7)	111 (98.2)	43 (100.0)	—	1.000^$^
MMF/MPA^@^	153 (98.0)	110 (99.7)	43 (100.0)	—	0.562^$^
Steroids	156 (100)	96 (85.0)	43 (100)		0.007^$^

*Symptoms*					
Fever	140 (89.7)	99 (87.9)	41 (95.3)	2.90 [0.63–3.3]	0.172
Cough	117 (75.0)	87 (77.0)	30 (69.8)	0.69 [0.32–1.51]	0.358
Sore Throat	53 (34.0)	35 (31.0)	18 (41.9)	1.61 [0.78–3.31]	0.204
Body Aches	77 (49.4)	59 (52.2)	18 (41.9)	0.66 [0.32–1.32]	0.247
Breathing Difficulty	48 (30.8)	24 (21.2)	24 (55.8)	4.68 [2.21–9.94]	0.001
Loss of Smell	22 (14.1)	21 (18.6)	1 (2.3)	0.10 [0.0014–0.80]	0.030
Distaste	36 (23.1)	33 (29.2)	3 (7.0)	0.18 [0.05–0.63]	0.003
Loose Motions	32 (20.5)	22 (19.5)	10 (23.3)	1.25 [0.54–2.92]	0.601
Extremes Weakness	9 (5.8)	6 (5.3)	3 (7.0)	1.34 [0.32–5.66]	0.691
Altered Sensorium	13 (8.3)	0 (0.0)	13 (30.2)	—	<0.001^$^

^*∗*^The odds ratio could not be computed due to zero count; ^$^Fisher's exact test. ^*@*^CAD/PVD, Coronary Artery Disease/Peripheral Vascular Disease; CNI, calcineurin inhibitors; Tac, Tacrolimus; CyA, CyclosporineA; MMF, Mycophenolate Mofetil; MPA, Mycophenolic Acid, #Fungal Culture Positivity when fungal infection was documented by positive urine or blood culture or Body Fluid Culture.

**Table 5:** Association between mortality and demographics, laboratory investigations, CT scan, and treatment options of KTRs with COVID-19.

Parameter	Survivors (*n* = 113)	Nonsurvivors (*n* = 43)	Mean/median difference (95% CI)	*p* value
*Demographics and baseline characteristics*				
Age (years), (Mean ± SD)	47.36 ± 13.28	55.02 ± 10.78	7.66 [3.56 to 11.76]	0.001
Height (meter) (Mean ± SD)	1.67 ± 089	1.66 ± 0.079	−0.012 [−0.043 to 0.0181]	0.428
Weight (kg) (Mean ± SD)	68.53 ± 14.58	69.91 ± 16.16	1.38 [−3.94 to 6.69]	0.610
BMI (kg/m^2^) (Mean ± SD)	24.45 ± 4.86	25.25 ± 5.34	0.80 [−0.96 to 2.57]	0.371
Transplant duration (Weeks) (Median[IQR])	256 [117–417]	327 [207–464]	71.0 [−1.86 to 146.14]	0.056

*Laboratory investigations (mean* *±* *SD)*				
Hemoglobin (gm %) (Hb)	11.86 ± 1.86 (*n* = 108)	10.26 ± 1.81 (*n* = 38)	−1.61 [−2.29 to −0.92]	<0.001
Total leucocyte count (cells/mm^3^)	8142 [6385–10300] *n* = 108	12200 [9028–16400] *n* = 37	3833 [2263–5540]	<0.001
Platelet count (×10^9^/L)	196.04 ± 65.84	148.6 ± 54.9	−47.45 [−69.42 to −23.49]	<0.001
Creatinine (mg/dL)	1.60 ± 0.89 (*n* = 106)	3.11 ± 1.89 [*n* = 38)	1.51 [0.87 to 2.15]	<0.001
Blood urea (mg/dL)	58.02 ± 25.0 (*n* = 105)	120.2 ± 60.44 (*n* = 35)	62.16 [40.9 to 83.45]	<0.001
Serum albumin (gm/dL)	3.81 ± 0.44 (*n* = 103)	3.12 ± 0.59 (*n* = 33)	−0.69 [−0.88 to −0.50]	<0.001
Lymphocytes (%)	14.79 ± 7.59 (*n* = 103)	11.94 ± 6.25 (*n* = 33)	−2.85 [−5.74 to 0.039]	0.053
Presentation SpO2 (%)	95.47 ± 3.36	87.74 ± 7.82	−7.73 [−9.49 to −5.96]	<0.001

*Inflammatory markers, (Median [IQR])*				
AST (IU/L)	28 [21–41] *N* = 96	32 [25–49] *N* = 34	5.5 [−4.74 to 11.50]	0.059
ALT (IU/L)	36 [20.6–54.5] *N* = 97	29 [19.3–49.0] *N* = 33	−2.86 [−11.0 to 5.50]	0.478
IL6 (pg/ml)	7.78 [2.70–28.15] *n* = 75	70.37 [31.22–199.75] (*n* = 33)	57.4 [34.3 to 103.1]	<0.001
Procalcitonin (ng/ml)	0.08 [0.04–0.24] *n* = 74	0.36 [0.11–2.74] *n* = 32	0.20 [0.08 to 0.51]	<0.001
D-dimmer (ngFEU/ml)	422.5 [287.9–881.3]	1212 [579–3540]	572.8 [285.0 to 1415.5]	<0.001
CRP (mg/L)	15.802 [2.66–50.94]	76.85 [34.30–126.60]	40.5 [25.6 to 66.4]	<0.001
Ferritin(ng/ml)	368.9 [93.4–1084.9]	962 [516–1889]	470.9 [147 to 794]	<0.001
LDH(IU/L)	292.5 [236.5–415.0]	437 [312–773.3]	136 [56.0 to 232.0]	<0.001

*AKI, dialysis and CT score, n(%)*				
AKI	29 (25.7)	36 (83.7)	14.90 [5.98 to 37.12]	<0.001
Need of dialysis	4 (3.5)	21 (48.8)	26.01 [8.13 to 83.24]	<0.001
CT score ≥ 15^&^	12 (31.6)	22 (75.9)	6.81 [2.29 to 20.28]	<0.001

*Treatment/Hospital management, n(%)*				
Remdesivir	15 (13.3)	30 (69.8)	15.08 [6.46 to35.20]	<0.001
Tocilizumab	7 (0.9)	9 (20.9)	29.65 [3.36 to242.4]	<0.001
Convalescent plasma	7 (6.2)	25 (58.1)	0.05 [0.02 to 0.13]	<0.001
Ventilator need	0 (0.0)	27 (62.8)	—	<0.001^$^
ICU stay	12 (10.6)	37 (86.0)	51.90 [18.17 to 148.3]	<0.001

^&^The number of subjects having CT scores was 67 (38 survivors and 29 nonsurvivors). ^$^ Odds ratio [95% confidence interval], ^$^Fisher's exact test. SpO2: Oxygen Saturation, Hb: hemoglobin, TLC: total leucocyte count, IL6: interleukin 6, LDH: lactate dehydrogenase, CRP: C-reactive protein.

**Table 6:** Multivariable logistic regression (MLR) analysis to evaluate independent effect of each biomarker on survivor status.

Test variable	Per unit	B(SE)	Odds ratio [95% CI]	*p* value	Number of cases
Mean IL6 (pg/mLl)^*∗*^	5 units	0.024 (0.011)	1.024 [1.003–1.047]	0.028	31 (NS) and 73(S)
Mean Procalcitonin (ng/mL)^*∗*^	0.1 units	0.001 (0.003)	0.999 [0.992–1.005]	0.744	29 (NS) and 72(S)
Mean D-dimer^*∗*^ (ngFEU/mL)	25 units (linear)	0.062 (0.025)	1.064 [1.013–1.117]	0.012	31 (NS) and 76 (S)
	(Quadratic)	0.00014 (0.00006)	1.00 [1.00–1.00]	0.013	
Mean CRP^*∗*^ (mg/L)	1 unit	0.004 (0.010)	1.004 [0.984–1.024]	0.689	31 (NS) and 71 (S)
Mean Ferritin (ng/mL)	25 unit	0.016 (0.013)	1.02 [0.99–1.043]	0.233	29 (NS) and 70 (S)
Peak LDH^*∗*^ (IU/L)	10 units	−0.006 (0.038)	0.994 [0.923–1.07]	0.872	25 (NS) and 66 (S)

^*∗*^The data has been adjusted for age, Hb, TLC, platelet count, blood urea, albumin level, fungal infection, chronic allograft dysfunction, and CAD/PVD. NS, nonsurvivor; S, survivor; IL6, interleukin 6; CRP, C-reactive protein; LDH, lactate dehydrogenase.

**Table 7:** Comparison of present work with similar studies conducted in KTRs infected with COVID-19 during the first and second epidemic waves.

Study Title⟶	Present work Jasuja et al.	Kute et al. [25]	Georgery et al. [47]	Elec et al. [21]	Villanego et al. [45]
*Study characteristics*					
Country	India	India	Belgium	East europe (Romania)	Spanish registry
Study design	Single-center	Single-center	Single-center	Single-center	Multicenter
Study period	1^st^ wave: 1^st^ February 2020–31^st^ January 2021	1^st^ wave: 15^th^ March–31^st^ December 2020	Not mentioned	1^st^ wave: March–September 2020	1^st^ wave: January-June 2020
	2^nd^ wave: 1^st^ March-31^st^ August 2021	2nd wave: 1^st^ April– 31^st^ May 2021.		2nd wave: October 2020–February 2021	2nd wave: July–December 2020
Number of subjects	1^st^ wave: 72	1^st^ wave: 157	1^st^ wave: 18	1^st^ wave: 33	1^st^ wave: 548
	2^nd^ wave: 84	2^nd^ wave: 102	2^nd^ wave: 27	2^nd^ wave: 149	2^nd^ wave: 463

*Demographics*					
Age	Comparable	More younger patients in 2^nd^ wave (study included pediatric population)	Comparable	Comparable	More younger patients 2^nd^ wave
Height	Comparable	Comparable	Comparable	Comparable	Comparable
Weight	Comparable	Comparable	Comparable	Comparable	Comparable
BMI	Comparable	Comparable	Comparable	Comparable	Comparable
Gender	Male predominance in both waves; comparable		Male predominance in the second wave	Male predominance observed in both waves	Male predominance observed in both waves; comparable
Comorbidities	Comparable between waves	More patients without comorbidities in the second wave	Patients from the second wave had more hypertension and multiple comorbidities	Comparable between waves	—
		More CMV coinfection and hypertension during 2^nd^ wave			
Time interval from transplantation to COVID-19 diagnosis	Comparable	Comparable	Comparable	Comparable	Comparable

*Baseline immunosuppression drugs*					
Steroids	Comparable	Comparable	—	Comparable	Comparable
CNI (Tac/CyA)	Comparable	More in the 1^st^ wave	—	Comparable	—
MMF/MPA	Comparable	More in the 1^st^ wave	—	Comparable	—

*Immunosuppressants modification during active COVID-19 disease*					
Antinucleoside/Antimetabolite drugs use	Stopped or reduced in most patients during both waves, comparable	Significantly stopped or tapered during the second wave	Stopped in both waves in all patients	Comparable	—
		(24.5% did not need any immunosuppressant modification during 2^nd^ wave)			
Steroids	In both waves, basal oral prednisolone was stepped up to 20 mg per day (any further modification was based on the patients' condition and appropriate recommendations)	Intravenous methylprednisolone is used in both waves more than in the first. Dexamethasone was the choice in the 2^nd^ wave	Increased use in 2^nd^ wave	Steroids were either kept at the maintenance dose or converted to IV for stress dosing in both waves	Increased use in the 2^nd^ wave
CNI	Altered (reduced or withheld) for more patients during the first wave	Not changed in most patients; comparable between waves	CNI reduced in all patients in both waves	Altered (reduced or withheld) for more patients during the first wave	—

*Striking symptoms difference observed between waves*					
COVID-19 basic symptoms	Symptoms including sore throat, body aches, loss of smell, distaste, loose motions, and running nose were reported significantly more frequently during the second wave	Milder symptoms such as cough were more frequent, while fever and expectoration were less reported symptoms during the second wave	Comparable symptoms	Disease severity was similar between the 2 waves	More patients were asymptomatic in the 2^nd^ wave
				More patients with COVID-19 pneumonia in the first wave	Fever, cough, lymphopenia, and incidence of pneumonia were less in the 2^nd^ wave
Mucormycosis	More cases during the second wave	More cases during the second wave	No mention	No mention	No mention
Allograft dysfunction	Comparable	More frequent in the second wave	—	—	—
AKI with or without dialysis requirement	Comparable	Higher in the second wave	—	—	—

*COVID-19 supportive/empirical management*					
Hospitalization	Less frequent during the second wave	Less frequent during the second wave	All patients were hospitalized	Less hospitalization during the second wave	Less hospitalization during the second wave
Doxycycline	Prescribed to more patients in the second wave	Less during the second wave	—	—	—
Tocilizumab	Prescribed to fewer patients in the second wave	Frequently used in the second wave	—	Comparable	Fewer patients in the 2^nd^ wave
Ivermectin	Prescribed to more patients in the second wave	Not used in the second wave (lack of evidence)	—	—	—
Remedisivir	Prescribed to fewer patients in the second wave	Frequently used in the second wave	—	Slightly more use in the 2^nd^ wave	More patients in the 2^nd^ wave
Azithromycin	Comparable	More frequent in the second wave		-	Less in the 2^nd^ wave
HCQS	Prescribed to fewer patients in the second wave	Frequently used in the second wave	None prescribed in the second wave	Minimal use in the second wave	Almost none (only one patient) prescribed in the 2^nd^ wave
Convalescent plasma	Prescribed to fewer patients in the second wave	Not used in the second wave (lack of evidence)	—	—	—
Favipiravir/fluvoxin/Ninitedanib	Prescribed to more patients in the second wave	Not used in the second wave (lack of evidence)	—	Slightly more use in the 2^nd^ wave	More use in the 2^nd^ wave
Antibiotics/antifungals	Prescribed to fewer patients in the second wave (antifungal use for mucor was more in the second wave)	Not used in the second wave (lack of evidence)	—	—	—
Thromboprophylaxis treatment	Prescribed to fewer patients in the second wave	Frequently used in the second wave	—	Less use in the 2^nd^ wave	-
ICU admission	Comparable	More during the second wave	Higher in the second wave	Comparable	Comparable
Ventilator	Comparable	Lesser patients in the second wave	—	Comparable	Slightly less during 2^nd^ wave (18% Vs 11%); statistically comparable
Oxygen requirement	Comparable	Lesser patients in the second wave	—	Comparable	—
CT scan	(i) Higher number of patients in the second wave	—	—	—	—
	(ii) More patients with severe CT scan scores in the second wave				

*Outcome and follow-up duration*					
Patient mortality rate	Overall patient mortality rate observed was 27.5%	1^st^ wave: 9.6%	1^st^ wave: 18.1%	1^st^ wave: 24.2%	1^st^ wave: 27.4%
		2^nd^ wave: 20%; comparable	2^nd^ wave: 27.2%; comparable	2^nd^ wave: 15.4%;	2^nd^ wave: 15.1%;
Follow-up timeline	1^st^ wave: 90 days	1^st^ wave: 28 days	1^st^ wave: 18 (5–30)	1^st^ wave: 60 days	—
	2^nd^ wave: 90 days	2^nd^ wave: 28 days	2^nd^ wave: 21 (6–40)	2^nd^ wave: 90 days	

BMI: Body Mass Index; CNI: calcineurin inhibitors; MMF: Mycophenolate Mofetil; Tac: Tacrolimus; CyA: cyclosporine A; HCQS: Hydroxychloroquine Sulfate; AKI: Acute Kidney Injury.

## Data Availability

All data used to support the findings of this study are available from the corresponding author upon request.
